# The effects of a combination of 3D virtual reality and hands-on horticultural activities on mastery, achievement motives, self-esteem, isolation and depression: a quasi-experimental study

**DOI:** 10.1186/s12877-022-03431-7

**Published:** 2022-09-12

**Authors:** Ching-Chih Fan, Cheuk-Sing Choy, Chiu-Mieh Huang, Po-Sheng Chih, Chia-Chiang Lee, Fen-He Lin, Jong-Long Guo

**Affiliations:** 1 Department of Community Medicine, En Chu Kong Hospital, Taipei, Taiwan; 2 Department of Nursing, Yuanpei University of Medical Technology, Hsinchu, Taiwan; 3 Institute of Clinical Nursing, College of Nursing, National Yang Ming Chiao Tung University, Taipei, Taiwan; 4 Department of Health Promotion and Health Education, College of Education, National Taiwan Normal University, No. 162, Sec. 1, He-ping East Road, Taipei, Taiwan; 5 Department of Nursing, College of Nursing, National Yang Ming Chiao Tung University, Taipei, Taiwan

**Keywords:** 3D virtual reality, Horticultural therapy, Community-dwelling, Older adults, Psychological well-being

## Abstract

**Background:**

Aging societies are a public health concern worldwide. It is critical to develop strategies that harness technology to enhance older adults’ mastery, achievement motives, self-esteem, isolation and depression effectively.

**Methods:**

This study aimed to explore the effects of a combination of three-dimensional virtual reality (VR) and hands-on horticultural activities on the psychological well-being of community-dwelling older adults. We used a quasi-experimental design. A total of 62 community-dwelling older adults were recruited and assigned to the experimental (*n* = 32) and comparison groups (*n* = 30). The members of the experimental group participated in an 8-week intervention program. Participants of both groups completed before-and-after intervention measurements for outcome variables that included perceived self-esteem, depression, isolation, and mastery and achievement motives, which were analyzed using the generalized estimating equation (GEE). A baseline score of depression was used as an adjustment for the GEE analyses to eliminate the effects of depression on outcomes.

**Results:**

After controlling age and gender as confounders, GEE analyses indicated that the experimental group showed significant post-intervention improvements in scores for self-esteem (β = 2.18, *P =* .005) and mastery (β = 1.23, *P =* .039), compared to the control group.

**Conclusions:**

This study supported a combination of three-dimensional VR and hands-on horticultural activities on community-dwelling older adults to improve self-esteem and mastery. The findings suggest that the future implementation of a similar program would be feasible and beneficial to community-dwelling older adults.

**Trial registration:**

The study was posted on www.clinicaltrials.gov (NCT05087654) on 21/10/2021. It was approved by the Institutional Review Board of En Chu Kong Hospital and performed in accordance with the Declaration of Helsinki.

## Background

Advances in healthcare technology have extended the average life expectancy of humans. The aging of populations has become a global public health issue. Older adults typically face a decline in their physical and psychological well-being in the final stage of their lives and often feel depressed and lonely due to the lack of interpersonal interaction after their retirement [1]. A previous large-scale survey indicated that 43% of people aged over 60 felt lonely [2]. Horticultural therapy is relatively suitable for older adults. A review examined the effectiveness of gardening programs, including 22 studies with various research designs. The findings revealed that gardening could promote overall health and quality of life, physical strength, fitness and flexibility, cognitive ability, and socialization [3]. Horticultural therapy also stimulates sensory functions, enabling older adults to achieve satisfaction and improve their self-esteem [3, 4]. Gardening activities often involve substantial interaction with peers, resulting in increased opportunities for social interaction and reduced depression and loneliness [5, 6]. Furthermore, older adults would experience a sense of achievement if they engage in horticultural therapy and successfully perform gardening activities.

To gain the benefits of horticultural therapy, older people need to learn the relevant skills and implement the learned skills. Motivation can enhance learning, and thus has an influence on performance [7]. As a result of advancing information and communication technology, virtual reality (VR) builds a virtual world with three-dimensional (3D) simulations. It can provide a virtual learning environment. 3D VR simulations provide an “immersive experience” that enables people to interact with virtual scenes and improve learning effectiveness [8]. A literature review discussed the features of immersive VR that provided an interactive human-computer interface with real-time simulation [9]. VR was suggested to be a promising tool for learning and training among older people. Therefore, integrating 3D VR into Horticultural therapy may enhance the motivation of older people and help them learn horticultural activities resulting in the promotion of hands-on performance. VR technology has been widely used in healthcare for the purposes of rehabilitation [10], pain management [11], cognitive training [12], and body function improvements [13, 14]. A previous study demonstrated that older adults have positive perceptions towards the acceptance of VR intervention [15]. For example, 30 older adults who underwent a 6-week VR program agreed that VR experiences were useful, easy to use, and pleasant [15]. In another study on a two-week VR intervention participants of the experimental group using the VR system reported being less socially isolated, being less likely to show signs of depression, and feeling better about their overall well-being, compared to the TV viewing group [16]. Previous research on the benefits of VR intervention has demonstrated a positive impact on the social well-being of older adults, particularly its potential to increase social interaction and provide a sense of accomplishment and improve mood [17]. VR possesses the potential to increase engagement between older adults and those around them, by providing topics of conversation [17]. Another study revealed that a combination of 3D VR and hands-on aromatherapy significantly improved the happiness, perceived stress, sleep quality, meditation experience, and life satisfaction in institutionalized older adults [18].

Previous studies have supported that horticultural therapy can improve self-esteem, reduce feelings of isolation and depression. In addition, the operation process of 3D VR can promote a sense of mastery and achievement among participants. This study applied a relatively new combination of 3D virtual reality and hands-on horticultural activities to design intervention programs. Therefore, we include self-esteem, depression, isolation, mastery and achievement as the outcomes of the intervention. The intervention study articulates the knowledge gap to contribute to the literature on feasibility and benefits of integrating technology into traditional horticultural activities. Thus, the study aimed to explore the effects of a combination of 3D VR and hands-on horticultural activities on community-dwelling older adults’ mastery, achievement motives, self-esteem, isolation and depression.

## Methods

### Study design and sampling

The study adopted a quasi-experimental design and was approved by the Research Ethics Review Committee of En Chu Kong Hospital (ECKIRB1090503). The study was conducted in 2 community elderly service centers in New Taipei City and was designated one facility as an experimental and the other as the comparison group. Participants were recruited via posters and verbal advertisements made by the staff during internal activities. All participants were willing to participate in the study and provided written informed consent. The selection criteria were as follows: older adults over 65 years old, intact cognition, the ability to understand verbal instruction and operate a VR joystick independently. The exclusion criteria included a history of hand dysfunction, severe visual and hearing impairment, being allergic to plants or pollen, and a current illness such as epilepsy or stroke.

### Sample estimation

Gpower 3.1 (HHU; Germany) software [19] was used to estimate sample size using an effect size of 0.7, an α error probability significant level set to 0.05 and power set to 0.8 and a statistical test with a means difference between 2 independent means; we found that a sample size of 52 was adequate. The effect size estimate of 0.7 was based on a previous similar study [20]. After considering a 20% attrition rate, we recruit 62 participants, 32 and 30 in the experimental and comparison group, respectively.

### Participants’ enrollment and assessment

**Fig. 1:**
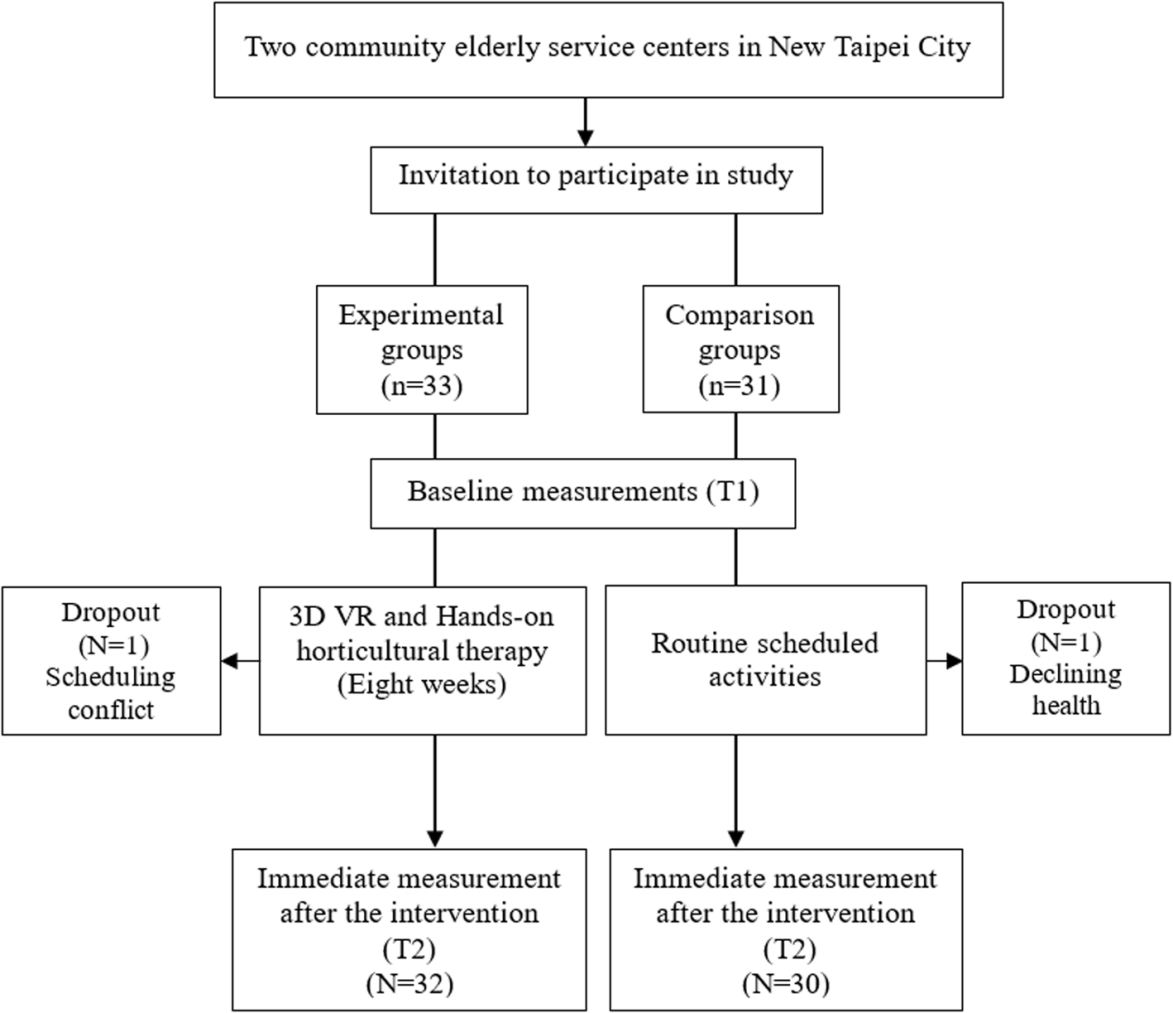
Enrollment and research process

The participants of the experimental and comparison groups are community-dwelling older adults living in an identical district with no significant group difference in the baseline characteristics. The enrollment and assessment process is shown in Fig. 1. After recruiting the 2 community elderly service centers, a research associate approached the executive director and staff to explain the purpose of the study and research procedure. After obtaining the administration’s consent, we distributed recruiting messages to potential participants and explained the informed consent form face-to-face. Each participant completed the consent form before the collection of baseline data, which was done one by one in a quiet and independent room at the facility. During the study period, 1 horticultural therapist, 2 VR operation instructors and 4 staff of the station were present to ensure the smooth progress of the study. In addition, we invited 4 older adults (2 males and 2 females) to examine the users’ experience of VR horticultural activities before the design of the study to confirm its feasibility for community-dwelling older adults. The experimental group participated in a gardening practice lesson designed by a horticultural therapist in coordination with relative VR themes. When the participants completed 3D VR horticultural activity, they obtained 2 virtual gold coins for each task. This feedback design enabled the participants to strive to complete the task to obtain rewards. The participants of the comparison group received scheduled activities, such as physical fitness, paper cutting, etc., without any gardening activities during the intervention and follow-up period.

### The combination of 3D VR and hands-on horticultural activities

The intervention program consisted of 8 two-hour sessions that were conducted once a week for 8 consecutive weeks, the program components are presented in Table 1. Before the intervention implementation, the research staff advised the participants on how to wear the VR helmet, operate the VR joystick and familiarized them with the VR scenes. The participants of the experimental group were divided into 4 groups during the weekly activities, and each group was assisted by facility staff. Before the activity, the staff explained the theme of the week and performed the hands-on horticultural activity after the VR operation.

**Table 1:**
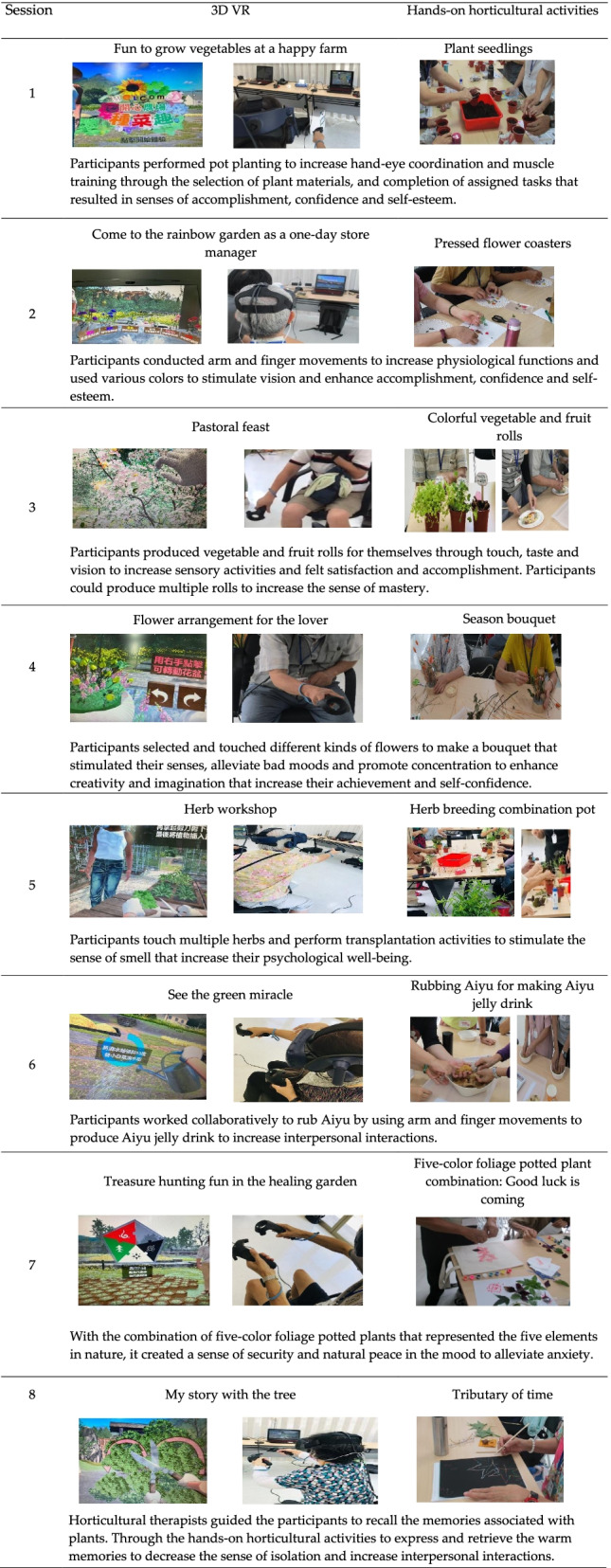
Program of the combination of 3D VR and hands-on horticultural activities

### Measurement instruments

Sociodemographic variables assessed at baseline are presented in Table 2.

**Table 2:** Baseline characteristics of participants

Variable	The experimental group (*n* = 32)		Comparison group (*n* = 30)		t/χ^2^	*p*
	Mean ± SD	n(%)	Mean ± SD	n(%)		
**Age**	70.94 ± 5.0		69.83 ± 3.8		t = 0.98	.129
**Gender**					χ^2^ = 2.39	.122
Male		8 (25.0)		3 (10.0)		
Female		24 (75.0)		27 (90.0)		
**Education**					χ^2^ = 9.01	.108
Illiteracy		1 (3.1)		3 (10.0)		
Primary		3 (9.4)		8 (26.7)		
Secondary		4 (12.5)		5 (16.7)		
High school		6 (18.8)		4 (13.3)		
University		18 (56.3)		10 (33.3)		
**Marital status**					χ^2^ = 5.86	.119
Married		19 (59.4)		20 (66.7)		
Widowed		5 (15.6)		8 (26.7)		
Divorced/separated		3 (9.4)		2 (6.7)		
Never married		5 (15.6)		0 (0.0)		
**Regular social events**					χ^2^ = 1.61	.204
No		7 (21.9)		3 (10.0)		
Yes		25 (78.1)		27 (90.0)		
**Chronic disease history**					χ^2^ = 0.26	.871
No		9 (28.1)		9 (30.0)		
Yes		23 (71.9)		21 (70.0)		

### Rosenberg self-esteem scale

This scale has been used previously in a study targeting older adults [21] and has good reliability and validity [22]. The scale consists of 14 items, each scored on a Likert-type scale that ranges from 1 (strongly agree) to 4 (strongly disagree), with higher scores indicating a higher level of self-esteem. A sample item is “Sometimes I do not think I have any merit at all.” The Cronbach’s α coefficients were 0.77 and 0.89 at baseline and posttest in this study.

### Geriatric depression scale-short form (GDS-15)

The Chinese version of the Geriatric Depression Scale [23] was used, which has a total of 15 items. All items were answered as either yes (1) or no (0). The total raw scores ranged from 0 to 15, with a higher score indicating a higher level of depression. Items 1, 5, 7, 11, and 13 were reverse scored because they are positive. The same Chinese version of this scale was used to study depression and its correlates among 195 older adults living in southern rural communities in Taiwan with a Cronbach’s α of 0.82 [24]. The Cronbach’s α coefficients were 0.82 and 0. 77 at baseline and posttest in this study.

### Perceived isolation

A short-form scale of perceived isolation was used to measure isolation. It consisted of 3 items as follows: 1. In general, how often do you feel that you lack companionship? 2. In general, how often do you feel left out? 3. In general, how often do you feel isolated from others? Each item was scored on a Likert-type scale from 1 (never) to 4 (often), with higher scores indicating a higher level of perceived isolation. The Cronbach’s α of the scale during its development was 0.70 [25]. The Cronbach’s α coefficients were 0.81 and 0.86 at baseline and posttest in this study.

### Perceived mastery

The Chinese version of the Perceived Mastery Scale [26] with a total of 7 items was used. Each item was scored on a Likert-type scale from 1 (strongly agree) to 4 (strongly disagree). The total raw score ranged from 7 to 28, with a higher score indicating a higher level of perceived mastery. A previous study indicated that Cronbach’s α coefficients were 0.82 [27]. The Cronbach’s α coefficients were 0.78 and 0.82 at baseline and posttest in this study.

### Achievement motives scale (AMS)

The AMS consists of 2 kinds of motives, referred to as Motive to Achieve Success, (MAS) and Motive to Avoid Failure (MAF) [28]. A short-form scale with 10 items was used to measure achievement motives, with 5 items each for MAS and MAF. Each item was scored on a Likert-type scale from 1 (strongly agree) to 4 (strongly disagree). A previous study indicated that Cronbach’s α coefficients were 0.88 and 0.86 at MAS and MAF, respectively [29]. The Cronbach’s α coefficients were 0.79 and 0.82 at MAS and MAF at baseline and posttest in this study.

### Interactive Q and A of horticultural knowledge

To examine learned horticultural knowledge among experimental participants, we designed interactive Q and A with 2 questions according to the learning contents of each VR session. The program consisted of 8 sessions, thus, participants completed 16 interactive questions during the VR program.

### Data analyses

Descriptive analyses were conducted for demographic and outcome variables. A 2-tailed t-test and χ^2^ test were used to compare differences in age, sex, education level, and chronic disease history between the experimental and comparison groups. A generalized estimating equation (GEE) was used to investigate the effect of time point, group, and their interaction on the outcome variables. GEE analyses enable an understanding on the patterns of change and their effects at both the individual and group levels [30]. Baseline scores of depression were used as an adjustment for the GEE analyses to eliminate the effects of depression on outcomes. Some studies suggest that age and gender were associated with learning outcomes [31–33]. For example, VR techniques can generally enhance participants’ learning outcomes which is affected by gender [31]. Another study found that age and gender were associated with learning and satisfaction [33]. In this study, there were no significant differences in age and gender between the two groups. However, differences in background between the two groups may not be detected due to the insufficient statistical power of a small sample size. Thus, GEE analysis included age and gender as confounding controls. Statistical analyses were conducted using SPSS (version 23.0; IBM Corp).

## Results

### Demographics

The participants’ average age was 70.94 (SD 5.0) years and 69.83 (SD 3.8) years in the experimental and comparison groups, respectively. There were no statistically significant differences in participants’ age (t = 0.98, *P* = .129), gender (χ^2^ = 2.39, *P* = .122), education level (χ^2^ = 9.01, *P* = .108), marital status (χ^2^ = 5.86, *P* = .119), regular social events (χ^2^ = 1.61, *P* = .204) and chronic disease history (χ^2^ = 0.26, *P* = .871) between the experimental and comparison groups (Table 2).

### Improvement of outcome variables

Comparison of 2 groups homogeneity by independent samples t-test, there were no significant differences in self-esteem (*P* = .528), depression (*P* = .156), isolation (*P* = .944), mastery (*P* = .822), and achievement motivation (*P* = .296) before intervention (Table 3).

**Table 3:** Summary of independent t-tests between experimental and comparison groups at baseline

Variable	Experimental group (*n* = 32)		Comparison group (*n* = 30)		*t*	*p*
	Mean	SD	Mean	SD		
Self-esteem	30.97	3.22	30.43	3.42	0.64	.528
Depression	2.88	2.64	4.08	3.27	−1.44	.156
Isolation	6.11	2.04	6.07	2.05	0.07	.944
Mastery	20.81	2.33	20.64	3.33	0.23	.822
Achievement Motives	3.40	3.16	2.50	3.44	1.06	.296

*p < .05, ** *p* < .01, ***p < .001

**Fig. 2:**
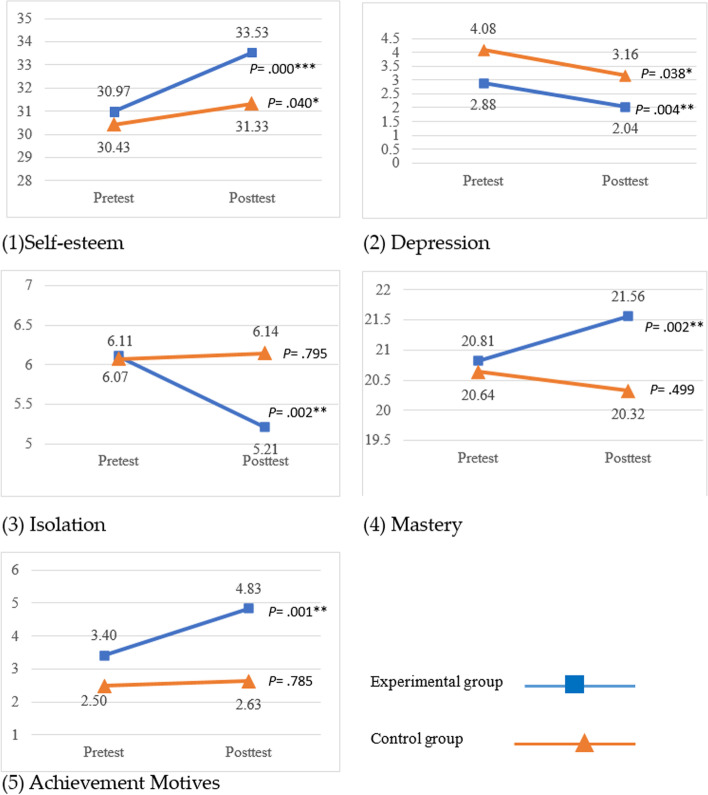
Changes in the pretests and posttests for 5 outcome variables between experimental and comparison groups. Higher scores indicate greater improvement in self-esteem, mastery, and achievement motives. Lower scores indicate greater reduction in depression and isolation

All of 5 psychological well-being variables including self-esteem (*P* < .001), depression (*P* = .004), isolation (*P* = .002), mastery (*P* = .002), and achievement motivation (*P* = .001) of experimental group had achieved significant improvement after intervention. The comparison group participated in 8 weeks of scheduled activities, with significant improvement in self-esteem (*P* = .040) and depression (*P* = .038) (Fig. 2).

The baseline scores for 5 outcome variables were not statistically significant between the experimental and comparison groups (Fig. 2). GEE analyses indicated that the experimental group showed significant post-intervention improvements compared to the comparison group for scores of self-esteem (β = 2.18, *P =* .005) and mastery (β = 1.23, *P =* .039) (Table 4).

**Table 4:** Results of GEE analyses on the 5 outcome variables

	GEE ^a^ coefficient (β)	SE	Wald χ^2^	*P-*value
**Self-esteem**				
Group (experimental group) ^b^	−0.04	0.84	0.02	0.963
Time (posttest) ^c^	0.63	0.41	2.31	0.129
Group (experimental group) X time (posttest) ^d^	2.18	0.77	7.96	0.005*
**Depression**				
Group (experimental group) ^b^	−0.15	0.14	1.21	0.272
Time (posttest) ^c^	−0.88	0.43	4.22	0.040
Group (experimental group) X time (posttest) ^d^	−0.02	0.50	0.002	0.963
**Isolation**				
Group (experimental group) ^b^	0.84	0.54	2.37	0.124
Time (posttest) ^c^	0.04	0.30	0.20	0.886
Group (experimental group) X time (posttest) ^d^	−0.82	0.43	3.73	0.053
**Mastery**				
Group (experimental group) ^b^	−0.62	0.75	0.68	0.411
Time (posttest) ^c^	−0.35	0.54	0.41	0.521
Group (experimental group) X time (posttest) ^d^	1.23	0.60	4.27	0.039*
**Achievement Motives**				
Group (experimental group) ^b^	0.75	0.99	0.58	0.447
Time (posttest) ^c^	0.21	0.49	0.18	0.668
Group (experimental group) X time (posttest) ^d^	1.15	0.66	3.00	0.083

Pretest score of depression was used as an adjustment for the GEE analyses. GEE analysis included age and gender as confounding controls

^a^*GEE* generalized estimating equation

^b^Reference group (group): comparison group

^c^Reference group (time): pretest

^d^Reference group (group time): comparison group pretest

3D VR was successful for the participants in learning horticultural knowledge. In the first 2 weeks of the program intervention, only one participant provided the wrong answer. From the third to the eighth weeks, all questions were answered correctly.

## Discussion

Previous studies had aimed to verify the impact of horticultural activities on the psychological well-being of older adults. In this study, we further combined 3D VR and hands-on horticultural activities to expand the intervention program, which is in line with the e-health trend. We found that this combination could promote the older adults’ self-esteem, mastery, and help them successfully obtain horticultural knowledge. The positive impact of horticultural activities on the mental health of older adults has also been supported by previous studies. For example, study participants’ self-esteem was significantly improved on operating community farms [34]. Another study revealed that community gardening is associated with resilience factors, self-esteem, optimism, and openness [35]. A study found that VR provides interactive learning and contributes to knowledge retention [36]. These findings are consistent with that of this study.

A previous study found that participants who used VR interactive scenarios reported improved perceived health and overall well-being, and reduced depression and social isolation relative to those in the comparison group (that only watched TV) [16]. However, the combination intervention did not improve the perceived depression and isolation of participants, which is inconsistent with previous studies. Researchers who conducted a 10-week indoor horticultural treatment activity for 10 older people (average age of 75.3 years) to evaluate the intervention effects of depression and loneliness, found that both had significantly improved (*P* < .001) [37]. A 3-year longitudinal study found that community-dwelling older adults who participated in domestic/gardening activities had a lower incidence of depression [38]. There were possible reasons why the intervention was ineffective on perceived isolation and depression among participants. First, the comparison group also participated in scheduled activities simultaneously, which may improve their perceived isolation and depression. Second, if any participant’s score in the GDS-15 (Chinese version) was less than 5 [39], it indicated the participant was not depressed. The GDS-15 is a self-assessment in reference to how they felt over the past week. A score ≥ 10 indicates a tendency of depression in general [39]. The percentages of participants whose scores were ≥ 10 in the experimental group and comparison group were 3.1 and 6.6%, respectively. The low percentages of depression status in the 2 groups suggest the reason why the intervention did not improve.

When participants used the 3D VR or conducted horticultural activities, our research team prepared various virtual and physical plants. Participants could independently select virtual plants to experience and then use the physical plants to make designed products. The horticultural therapist and staff guided and assisted participants in finishing their products. The participants can perform the horticultural skills practiced in the VR environment by hand actually. The participants presented their horticultural products to each other and shared their feelings. The learning processes enhanced their senses of mastery. According to Bandura’s self-efficacy model, having a direct experience of mastery is an important source of increasing self-efficacy [40]. High self-efficacy would contribute to better performance which is associated with self-esteem. We suggest that future research could adopt similar designs using horticultural activities that allow the older adults to independently select and operate horticultural material, and to share their work after completion to strengthen their perception of finishing the designed activities, to further improve perceived mastery.

Our study revealed that community-dwelling older adults could successfully complete an 8-week VR horticultural program after practicing with the assistance of the research team. It was consistent with that of a previous study [41]. It examined 10 community-dwelling older adults without previous user experience of VR in a semi-structured interview and 2 subsequent focus group sessions about their perceptions about using VR devices. VR was feasible for use by older adults, even for those who were immobile or resided within care facilities.

Our study had several limitations. First, the intervention sites of this study are community elderly service centers. Our findings may not be considered generalizable to residents in long-term care facilities. Second, owing to the coronavirus disease 2019 pandemic, all participants strictly wore masks during the intervention period, which prevented them from smelling the plants used in the program. Future studies with similar intervention programs should use modified masks that are better facilitate breathing to increase the intervention effects. Third, the relatively low percentages of the presence of depression in the baseline of the experimental and comparison groups decreased the intervention effect on the depression variable. Lastly, the relationship between cognitive function and depressive symptoms has been a topic of discussion for a long time; measurement methods, such as mini-mental state examination scores, could be used to evaluate the cognitive function of the participants.

## Conclusion

This study supported a combination of three-dimensional VR and hands-on horticultural activities on community-dwelling older adults to improve self-esteem and mastery. The findings suggest that the future implementation of a similar program would be feasible and beneficial to community-dwelling older adults.

## Data Availability

The datasets used and analyzed during the current study are available from the corresponding. Author on reasonable request.

## Abbreviations

3D VR: Three-dimensional virtual reality
GEE: Generalized estimating equation
GDS-15: Geriatric depression scale-short form
AMS: Achievement motives scale
MAF: Motive to avoid failure
